# Acute abdomen due to primary omentitis: a case report

**DOI:** 10.1186/1477-7800-4-19

**Published:** 2007-07-26

**Authors:** Michael Safioleas, Michael Stamatakos, Konstantinos Giaslakiotis, Anastasios Smirnis, Panagiotis Safioleas

**Affiliations:** 1 2nd Department of Propedeutic Surgery, School of Medicine, Athens University, Laiko General Hospital, Greece; 2 Department of Pathology, School of Medicine, Athens University, Greece

## Abstract

**Background:**

Idiopathic segmental infarction of the greater omentum (ISIGO) is an uncommon cause of acute abdomen in children and adults and its etiology is rather vague and speculative. The clinical presentation is usually with atypical acute or subacute abdominal pain. In a number of cases radiologic imaging allows proper preoperative diagnosis and treatment.

**Case presentation:**

We report a case of ISIGO in a 31 year old patient, who presented with acute abdominal pain, nausea, vomiting and leukocytosis. Radiologic investigation was non-specific. The patient underwent surgical resection of the infracted omentum with compete recovery.

**Conclusion:**

ISIGO should be considered in the differential of acute abdomen especially when presentation is atypical and all other causes have been excluded. In cases with non-specific radiologic findings, laparotomy is necessary for proper diagnosis and treatment. Surgical resection of the infracted omentum results in uneventful recovery in the majority of cases.

## Background

Primary or idiopathic segmental infarction of the greater omentum (ISIGO) is an uncommon cause of acute abdomen in adults and children and has a vague and rather speculative etiology [1-3]. Less than 400 cases have been published in the English literature, since the first description by Bush in 1896 and its incidence is probably underestimated [4]. Omental infarction secondary to associated intra-abdominal conditions is in contrast a rather familiar event [5]. The majority of patients with primary or secondary omental infarction present with non specific symptoms of abdominal pain and discomfort and despite the usefulness of abdominal ultrasound (US) and computer tomography (CT), the diagnosis is in many cases elusive preoperatively [6]. Resection of the infracted omentum during laparotomy or laparoscopically, results in full recovery in the majority of cases [6,7]. We report a young male patient with primary segmental infarction of the greater omentum, presented as acute abdomen.

## Case presentation

A 31-year-old man was admitted to our department because of acute abdominal pain, nausea, vomiting and anxiety of ten hours duration. On physical examination severe abdominal tenderness was marked, localized to the right lower abdomen aggravated on walking and coughing. His temperature was 37.8 degrees celcius and his pulse rate 92 bpm. Laboratory investigation disclosed a white blood count of 16,800/mm3, neutrofils 72% with left shift and elevated erythrocyte sedimentation rate (ESR) and C reactive protein (CRP).

**Figure 1:**
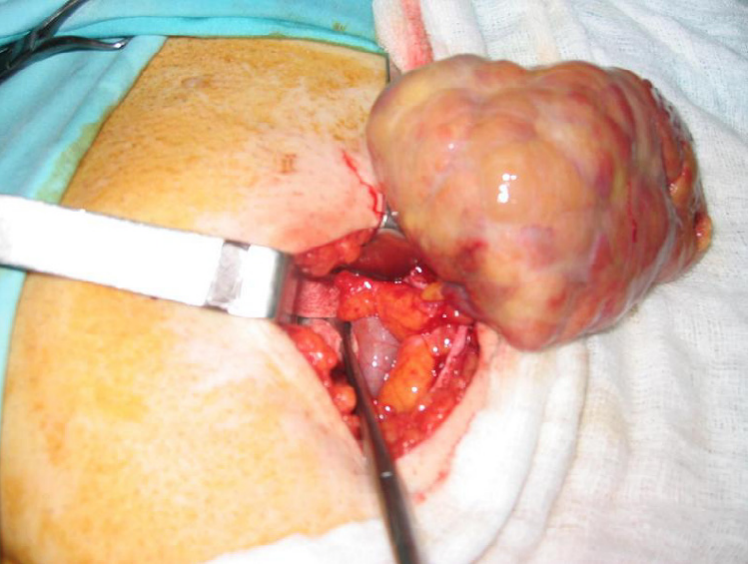
Omental inflammatory mass.

All other hematological and biochemical parameters were within normal range. Clinical presentation and laboratory findings suggested acute appendicitis and laparotomy was performed. Through a right paramedian subumbilical incision the right iliac fossa was explored and a grossly inflamed omental mass was revealed and resected. (Fig. 1).

A small amount of fluid was seen. The appendix was macroscopically normal and no other cause of acute abdomen was identified. The pathology of the surgical specimen reported primary omentitis. The patient made an uneventful recovery.

## Discussion

Segmental infarction of the large omentum is an uncommon condition that may present as acute abdomen in any age group, mainly in the fourth and fifth decade of life, with a male to female ratio of 2:1 [6]. It is seldom considered in the differential diagnosis of acute abdomen and its incidence may be underestimated. From a pathogenic point of view omental infarction is classified as secondary when it occurs in association with coexisting intraabdominal pathologies, such as internal or external hernias, chronic inflammatory conditions, adhesions and neoplasias and as primary or idiopathic when no other pathologic condition is identified. The latter is less common and has a rather vague etiology. Local omental abnormalities, such as anatomical malformations, redundant omental veins, local variations in omental fat distribution, leading to venous stasis and thrombosis have been suggested in the pathogenesis but the etiology is unclear [1,3,6].

Although controversial, as has been suggested but not proven, an infectious cause has also been reported for the spontaneous segmental infarction of the greater omentum [3,8].

In the majority of the published cases, omental infarction is located to the right side probably due to the greater size and mobility of the right sided omentum in relation to the left side [9]. Precipitating factors comprise trauma, increased intra-abdominal pressure following coughing, exertion, heavy meal and change in body position, which probably result in sudden misplacement of the omentum and compromised blood flow [6].

Clinical presentation is generally atypical. Cardinal manifestations are acute or subacute abdominal pain, commonly of right lower quadrant or right paraumbilical in location and severe abdominal tenderness, which is no radiating and progressively increasing in severity. Fever is usually absent or of low grade. In general, there is no nausea, vomiting, anorexia or other gastrointestinal complain. Laboratory investigation discloses mild leukocytosis and elevated ESR and CRP [1,3]. Abdominal US and CT scan reveal, in the majority of cases, solid hyperechoic lesions and a whirling mass with hyper-attenuated fatty tissue, respectively [10,11]. The presence of concentric linear strands in abdominal CT scan is highly characteristic of omental infarction and it allows accurate preoperative diagnosis [11]. Nevertheless, in many patients surgical intervention is necessary for definite diagnosis and treatment [6].

Laparotomy or laparoscopy discloses a fatty hemorrhagic mass in the abdominal cavity accompanied by free serosanguineous fluid [6,12]. Careful surgical exploration of the appendix, ovaries and other intra-abdominal organs is warranted. By definition, no accompanied intra-abdominal pathology is present.

Resection of the infracted omentum during laparotomy or laparoscopically is considered to be mandatory. Nevertheless, there are reports of effective non-operative treatment of omental torsion and segmental infarction of the greater omentum correctly diagnosed by imaging techniques, when the patient's condition is stable. The authors suggest that in these uncomplicated cases conservative treatment is acceptable [6]. However, surgical intervention is associated with faster recovery, better pain control and prevents complications, such as adhesion, stricture formation, bowel obstruction, abscess formation and possibly sepsis [12]. In our case, atypical clinical presentation and unspecific laboratory and radiological findings resulted in laparotomy, which disclosed idiopathic omental infarction. Surgical resection of the infracted omentum resulted in uneventful recovery.

## Conclusion

In conclusion, ISIGO should be considered in the differential of acute abdomen, especially in cases with atypical presentation. Abdominal CT scan may reveal the underlying pathology. However, in a number of cases surgical intervention is necessary for definite diagnosis. Resection of the infracted omentum during laparotomy or laparoscopically is the treatment of choice, prevents future complications and is associated with fast recovery.

## Abbreviations

ISIGO: idiopathic segmental infarction of the greater omentum;

US: ultrasound;

CT: computer tomography;

ESR: erythrocyte sedimentation rate;

CRP: C-reactive protein

## Competing interests

The author(s) declare that they have no competing interests.

## Authors' contributions

MS: Surgeon who performed the operation and edited a part of the manuscript.

MS: Surgeon who performed the operation and prepared the draft.

KG: Doctor who performed the pathological examination and edited part of the manuscript.

AS: Surgeon who contributed to the performance of the operation.

PS: Literature search, revision of bibliography.

All authors have read and approved the final manuscript.
